# Study on Mechanical Properties of Adjustable-Ring-Mode Laser Scanning Welding of TA1 Titanium Alloy to 304 Stainless Steel Dissimilar Thin Sheets

**DOI:** 10.3390/ma19020230

**Published:** 2026-01-07

**Authors:** Geng Li, Tengyi Yu, Peiqing Yang, Suning Zhao, Shuai Zhang, Honghua Ma, Shang Wu, Ji Li, Ming Gao

**Affiliations:** 1 School of Physics and Electronic-Information Engineering, Hubei Engineering University, Xiaogan 432000, China; 2 Wuhan National Laboratory for Optoelectronics, Huazhong University of Science and Technology, Wuhan 430074, China; 3 State Key Laboratory of Vanadium and Titanium Resources Comprehensive Utilization, Pangang Group Research Institute Co., Ltd., Panzhihua 617000, China; 4 School of Mechanical Engineering, Hubei University of Technology, Wuhan 430068, China

**Keywords:** adjustable-ring-mode laser, scanning welding, TA1/SS304 dissimilar thin sheets, mechanical properties

## Abstract

The adjustable-ring-mode (ARM) scanning laser was used to perform butt welding on 0.5 mm thick TA1 titanium alloy and 304 stainless steel (SS304) thin sheets, with 1.2 mm diameter AZ61S magnesium alloy welding wire as the filling material. Microhardness test results show that the hardness distribution presented a trend of being higher in the base metals on both sides and lower in the middle filling area, with no hardening observed in the weld zone. For all specimens subjected to horizontal and axial weld bending tests, the bending angle reached 108° without any cracks occurring. When the ring power was in the range of 800–1000 W, or the scanning frequency was between 100 and 200 Hz, all the average tensile strengths of the welded joints were more than 80% of that of the AZ61S filling material (approximately 240 MPa); the maximum average tensile strength stood at 281.2 MPa, which is equivalent to 93.7% of the AZ61S. As the ring power or scanning frequency increased further, the tensile strengths of the joints showed a decreasing trend. The remelting effect of the trailing edge of the ARM laser under high energy conditions, or the scouring of the turbulent molten flow induced by the scanning beam, damages the weak links at the newly formed solid–liquid interface and increases the Fe concentration in the molten pool. This leads to a thicker FeAl interface layer during growth, thereby resulting in a decline in the mechanical properties of the welded joints.

## 1. Introduction

Titanium and its alloys are characterized by high specific strength, excellent resistance to elevated temperatures and corrosion, as well as good biocompatibility. These properties enable their extensive application in automotive, aerospace, offshore engineering equipment, and biomedical fields [1]. However, their utilization is often constrained by high production costs and limited machinability. In contrast, stainless steel possesses advantages such as favorable mechanical properties, superior processability and weldability, along with lower production and usage costs, making it one of the most widely used structural materials today [2]. Nevertheless, stainless steel exhibits inferior high-temperature mechanical properties and corrosion resistance compared to titanium alloys, along with higher material density, which also restricts its applications. Consequently, effectively combining titanium alloys and stainless steel can integrate the merits of both—the high specific strength and excellent corrosion resistance of titanium alloys with the cost-effectiveness of stainless steel. This synergy fully addresses the requirements for structural weight reduction and functional diversification in modern high-end manufacturing, which demonstrates promising application prospects.

Currently, the joining of thin titanium-to-steel sheets primarily employs several welding methods, including solid-phase welding (predominantly explosion welding, diffusion welding, and friction stir welding), fusion welding (primarily laser welding), and brazing. Each of these approaches has distinct advantages and disadvantages. Explosion welding is characterized by difficulties in precisely controlling the welding process, severe interface deformation, and non-uniform interfacial bonding strength coupled with poor joint quality stability resulting from uncontrollable explosive wave-induced deformation; it also retains significant residual stress, which requires subsequent annealing treatment [3]. Although diffusion welding yields good weld quality, it suffers from lengthy temperature control periods, low efficiency, and stringent environmental requirements, rendering it largely impractical under most industrial conditions [4]. While friction stir welding features a small heat-affected zone, its production efficiency is relatively low; furthermore, the intense mechanical action of the stirring pin causes pronounced migration of Fe elements into the titanium side, forming alternating layers rich in Fe and Ti, which consequently renders the joint prone to fracture under load [5]. The quality of brazing is significantly influenced by the composition of the filler material, while the joint strength is highly sensitive to the width of the joint gap and surface cleanliness; furthermore, constrained by overall heating and vacuum environment requirements, brazing also cannot achieve high production efficiency [6].

Unlike the aforementioned welding methods, laser welding exhibits simpler procedures, good flexibility, and high efficiency, which makes it more suitable for large-scale industrial applications [7]. Although laser welding has broad application prospects in joining titanium–steel thin sheets, its keyhole effect inherently manifests the characteristic of deep penetration welding. For thin plates with thicknesses no more than 1 mm, laser welding possesses a narrow process window and is highly prone to instability during the process, resulting in significant fluctuations in penetration depth and bead width—or even burn-through, causing extensive porosity.

To address the issues mentioned above, the introduction of scanning during the welding process can be considered. Scanning laser welding offers significant advantages in improving the distribution of laser energy and enhancing weld quality. To suppress porosity caused by keyholes in Al-Mg alloy laser welding, Xu developed high-frequency outwards-spiral scanning laser welding, wherein the laser beam reciprocates within a single keyhole. The keyhole collapse frequency decreased from 137.8 Hz in conventional welding to 8.5 Hz, while the tensile strength reached 371 ± 2 MPa, representing a 21.0% improvement [8]. To suppress spatters, Mei et al. introduced scanning methods and investigated the effects of different scanning trajectories on the stability of T2 copper laser welding. When employing “O”-shaped, “8”-shaped, and “infinity”-shaped scanning paths, the keyhole variation period was prolonged, the welding stability was improved, and the spatters were effectively suppressed. Among all these scanning paths, the “O”-shaped scanning demonstrated the best spatter suppression effect, with a spatter quantity reduction of 48.8%. The maximum joint tensile strength reached 234 MPa, achieving 97.5% of the base material strength. The porosity was minimized to 1.33%, only 38% of that in conventional laser welding [9]. They further proposed a magnetic field-assisted scanning laser welding technique to address the low energy coupling efficiency and poor process stability in T2 copper laser welding. Under a 120 mT magnetic field, metal vapor and plasma were minimized, spatters were eliminated, and the weld formation was uniform without significant defects. The maximum joint tensile strength reached 202 MPa, representing a 14.8% increase compared to non-magnetic conditions [10].

Hui and Chen investigated the effects of scanning laser welding on molten pool flow and weld formation in 304L stainless steel corner joints, as well as on the microstructure and mechanical properties of 6061/2A12 dissimilar aluminum alloy welded joints [11,12]. For 304L stainless steel, the recommended scanning frequency is below 200 Hz and the scanning amplitude below 2 mm to prevent undercut at the weld root. For 6061/2A12 aluminum alloy, increasing the laser scanning frequency and amplitude increases the proportion of equiaxed crystals in the fusion zone and reduces their average diameter. The fracture location shifts from the fusion zone to the heat-affected zone, and the elongation of tensile specimens increases by 120% compared to non-scanned welds. The above research results indicate that scanning laser welding is suitable for welding various metals, but its application in thin sheet welding remains challenging. This is primarily because the light source is mostly a continuous laser with a classical Gaussian beam mode, which inherently suffers from excessive central energy concentration. Consequently, defects such as undercuts and spatters are prone to occur. To address this, improvements are considered at the welding heat source end by introducing a more energy-dispersed adjustable-ring-mode (ARM) laser.

The ARM laser is a composite light source formed by superimposing an annular auxiliary light source outside a conventional point light source, enabling independent regulation of point/ring illumination. Li et al. noted that the ARM laser welding of aluminum alloys achieves effective suppression of spatters. The outside ring laser preheats the leading edge of the molten pool, prevents abrupt fluctuations in laser absorptivity, stabilizes metal vapor ejection, and thereby significantly mitigates spatter formation [13]. In addition, compared with traditional Gaussian lasers, the ARM laser can effectively mitigate porosity in aluminum alloy welding. For instance, as the ARM laser power increases, the movement speed of bubbles at the keyhole bottom decreases by 63.2%, enhancing their probability of being captured by the keyhole and eventually vanishing [14]. Wang’s research also indicated that using an ARM laser in aluminum alloy welding not only improves welding process stability but also achieves higher weld surface quality [15]. Tang attempted to use the ARM laser and scanning motion in aluminum alloy welding, and found the maximum deformation in the Z-direction is 1.403 mm, which is a reduction of 1.702 mm compared to the non-ARM laser [16]. Xu used an ARM laser on the welding of Al-Mg-Er-Zr alloy to suppress material segregation, and achieved a uniform temperature distribution and a fully equiaxed microstructure. Tensile strength of the welded joints reaches 389 ± 1 MPa, which is 93.3% of the base material [17].

Except for the above mechanism analysis of ARM laser welding, regarding applicable materials, Weiss used an ARM laser to weld 80 μm AISI 316L stainless steel foils with a maximum welding speed of 920 mm/s [18]. Liu employed an ARM laser to join continuous carbon fiber reinforced PEEK and 2024 aluminum alloy composite material, and found that a joint temperature of 750 K results in better lap shear strength [19]. The above studies show that compared to Gaussian lasers, the ARM laser significantly increases welding process stability, reduces porosity, leads to less deformation, and provides better mechanical properties. At the same time, due to its larger spot size, the ARM laser has better bridging ability for joint gaps and higher tolerance for misalignment than Gaussian lasers. Therefore, the ARM laser is very suitable for thin sheet welding.

For the titanium-steel welding involved in this paper, and even all dissimilar metal welding, the mechanical properties of the welds are generally low. The ARM laser could have played a significant role; regrettably, there are few reports on this aspect at present, let alone the incorporation of scanning technology. Therefore, this paper innovatively proposes to use the ARM scanning laser for welding titanium-steel dissimilar metal thin sheets and focuses on studying the mechanical properties of the welds.

## 2. Materials and Methods

TA1 titanium alloy and 304 stainless steel (SS304) with a thickness of 0.5 mm were used as the base metals, respectively. Before welding, the workpieces were processed into a size of 100 mm × 50 mm. AZ61S magnesium-alloy welding wire with a diameter of 1.2 mm was used as the filling material. According to GB/T 3620.1-2016, GB/T 1220-2007, and GB/T 5153-2016, the chemical compositions of the base metals and filling material are presented in Table 1. In accordance with the materials handbooks compiled by Song, Yan, and Zeng et al. [20,21,22], as well as GJB 3321-1998, the mechanical properties of each material are listed in Table 2.

A DGLaser DGMM2000/2000-APP ARM laser (Sichuan DG Laser Technologies Co., Ltd., Mianyang, China) was adopted, with a maximum central power of 2000 W, a maximum ring power of 2000 W, and a maximum total power of 4000 W. A Fronius VR7000-CMT wire feeder (Fronius International, Wels, Austria) was used, featuring an adjustable wire feeding speed range of 0.5–22 m/min. The motion device was a Fanuc 6-degree-of-freedom robot (Shanghai Fanuc Robotics Co., Ltd., Shanghai, China), which realized the circular scanning motion of the laser through a scanner. In the experiment, a butt joint was used, with a gap of 0.4 mm between the two plates. Under argon protection, wire-filled welding was performed using the ARM scanning laser.

The parameters of the ARM laser included central power and ring power. Since both the central laser and the traditional non-ARM laser are composed of Gaussian beams, the most significant difference between the ARM laser and the non-ARM laser lies in the magnitude of the ring power. Similarly, the parameters of the scanning laser included scanning frequency and scanning amplitude. Due to the relatively small scanning amplitude, the key distinction between the scanning laser and the non-scanning laser is the scanning frequency.

**Figure 1:**
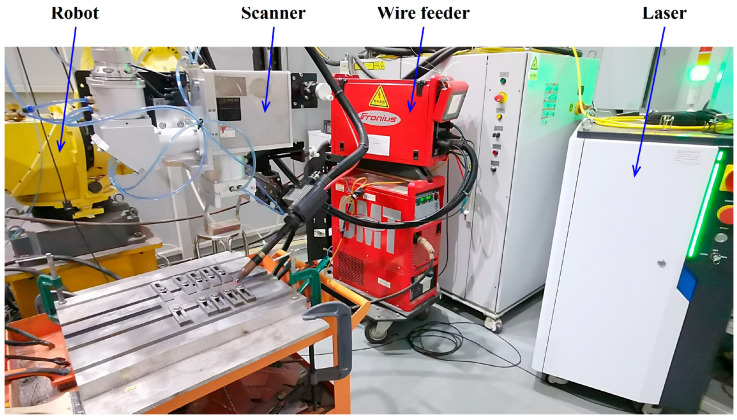
Experimental setup.

In this paper, the effects of ring power (as a representative parameter of the ARM laser) and scanning frequency (as a representative parameter of the scanning laser) on the mechanical properties of the welded joints were systematically investigated. During the experiment, only one parameter was changed at a time, while other parameters were kept constant. To ensure that the thin sheets were fully penetrated without burn-through defects and that the welded material would not undergo brittle fracture when slightly touched, the relatively reasonable variation ranges of each process parameter and the baseline values for fixed parameters were determined through preliminary experiments, as shown in Table 3. The experimental setup is illustrated in Figure 1.

After welding, microhardness specimens were prepared following standard procedures and were polished and etched. A VEIYEE HV-1000TPTA Vickers hardness tester (Laizhou Veiyee Test Instrument Manufacturing Co., Ltd., Laizhou, China) was used to conduct microhardness tests in accordance with ISO 6507-1:2023 [23]. During the tests, a load force of 200 g was applied with a dwell time of 15 s, and the spacing between adjacent test points was 0.2 mm. A SHIMADZU AG-IC electronic universal testing machine (Shimadzu Corporation, Kyoto, Japan) was employed to perform bending tests on the welded joints at a loading rate of 0.5 mm/min, in compliance with ISO 5173:2023 [24]. The diameter of the bending indenter was 30 mm, and the specimen size was 100 mm × 8 mm; the test categories included horizontal weld bending and axial weld bending. The same SHIMADZU AG-IC electronic universal testing machine (Shimadzu Corporation, Kyoto, Japan) was used for tensile tests on the welded joints at a loading rate of 0.5 mm/min, adhering to ISO 4136:2022 [25]. The number of bending specimens and tensile specimens was three each, and the average value of the test results from the three specimens was calculated. The fracture morphology of the tensile specimens was observed using a scanning electron microscope (SEM), and the chemical composition of typical fracture regions was measured via energy-dispersive spectroscopy (EDS) analysis.

## 3. Results

### 3.1. Microhardness

**Figure 2:**
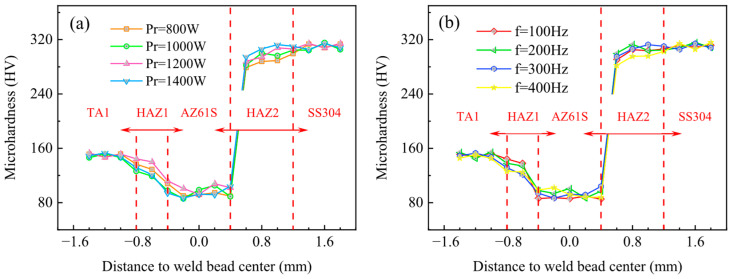
Effects of process parameters on the microhardness of welded joints: (**a**) ring power (when f = 300 Hz) and (**b**) scanning frequency (when P_r_ = 1400 W).

The effects of different ring powers and scanning frequencies on microhardness of the welded joints were measured, respectively, with typical results shown in Figure 2. From left to right, the regions are sequentially the base metal region (TA1), the heat-affected zone between TA1 and the weld region (HAZ1), the weld region (AZ61S), the heat-affected zone between the weld region and SS304 (HAZ2), and the base metal region on the other side (SS304). Uncertainty analysis was performed on the measurement results in accordance with ISO 6507-1:2023, ISO 6507-2:2018 [26], and ISO 6507-3:2018 [27]. In the first step, a measurement model was established. The measurement model for Vickers hardness is as follows:

(1) $$HV=0.1891\frac{F}{d^{2}}$$

where *F* represents the test force, and *d* represents the arithmetic mean of the lengths of the two indentation diagonals. In the second step, the sources of measurement uncertainty were analyzed. These mainly include the following uncertainty components: *u*(*d*), the uncertainty component caused by the measurement repeatability of the indentation diagonal length and the measurement resolution of the indentation diagonal length; *u*(*F*), the uncertainty component caused by the measurement error of the test force *F*; *u*(*b*), the uncertainty component caused by the uniformity of the standard hardness block; *u*(*t*), the uncertainty component caused by the indication error of the hardness tester; and *u*(*r*), the uncertainty component caused by the numerical rounding of the measurement results.

In the third step, the standard uncertainty components were evaluated. Since the hardness values corresponding to different experimental parameters vary—particularly in the weld zone and heat-affected zone—the hardness measurement results under the typical parameters of P_r_ = 1400 W and f = 300 Hz were used as an example for illustration here. First, the uncertainty component *u*_1_(*d*) caused by the measurement repeatability of the indentation diagonal length *d* was calculated using the formula:

(2) $$u_{1}\left(d\right)=\sqrt{\sum_{i=1}^{n}{\left(d_{i}-\bar{d}\right)}^{2}/\left(n-1\right)}$$

where *d_i_* is the average length of the two indentation diagonals in the *i*-th test, $\bar{d}$ is the total average of the indentation diagonal lengths, and *n* is the total number of measurements. For the five regions in Figure 2 (TA1, HAZ1, AZ61S, HAZ2, and SS304), the values of *u*_1_(*d*) are 0.000503 mm, 0.002 mm, 0.002035 mm, 0.000432 mm, and 0.000208 mm, respectively. Second, the uncertainty component *u*_2_(*d*) caused by the measurement resolution of the indentation diagonal length was calculated. According to JCGM 100:2008, for a digital-display measuring instrument, if the resolution is *δ_x_*, the uncertainty caused by the measurement resolution is *u*(*x_i_*) = 0.29*δ_x_*. The measurement resolution of this hardness tester is 0.0001 mm, so *u*_2_(*d*) = 0.000029 mm. The combined uncertainty component *u*(*d*) of the indentation diagonal length *d* was obtained by combining *u*_1_(*d*) and *u*_2_(*d*) using the formula:

(3) $$u\left(d\right)=\sqrt{u_{1}{\left(d\right)}^{2}+u_{2}{\left(d\right)}^{2}}$$

where for TA1, HAZ1, AZ61S, HAZ2, and SS304, the corresponding *u*(*d*) values are 0.000504 mm, 0.001697 mm, 0.002035 mm, 0.000433 mm, and 0.00021 mm, respectively.

Next, the uncertainty component *u*(*F*) caused by the measurement error of the test force *F* was calculated. According to the verification certificate of the hardness tester, the measurement error of the test force *F* obtained via testing with a standard dynamometer is 0.1%. The uncertainty caused by this error is considered to follow a uniform distribution, calculated using the formula:

(4) $$u\left(F\right)=a_{F}/\sqrt{3}$$

where *a_F_* is the half-width of the measurement error of the test force. Here, the test force measured by HV0.2 is 1.961 N, and *u*(*F*) is calculated to be 0.000566 N. Similarly, the uncertainty component *u*(*b*) caused by the uniformity of the standard hardness block can be expressed as

(5) $$u\left(b\right)=a_{b}/\sqrt{3}$$

where *a_b_* is the half-width of the uniformity of the standard hardness block. According to the verification certificate of the 475 HV0.2 standard hardness block, the uniformity of this standard block is 0.5 HV, and *u*(*b*) is calculated to be 0.144337 HV. The uncertainty component *u*(*t*) caused by the indication error of the hardness tester can be expressed as

(6) $$u\left(t\right)=a_{t}/\sqrt{3}$$

where *a_t_* is the half-width of the measurement error of the average hardness value. It is known from the verification certificate of the hardness tester that the indication error of the hardness tester is 0.8%. The calculated *u*(*t*) values for the five regions (TA1, HAZ1, AZ61S, HAZ2, and SS304) are 0.345794 HV, 0.291908 HV, 0.216483 HV, 0.706908 HV, and 0.714067 HV, respectively. Finally, the uncertainty component *u*(*r*) caused by the numerical rounding of the measurement results was calculated. According to JCGM 100:2008, when rounding a measured value, if the rounding interval is *δ_x_*, the uncertainty caused by numerical rounding is *u*(*x_i_*) = 0.29*δ_x_*. Here, *δ_x_* = 1 HV, so *u*(*r*) is 0.29 HV.

In the fourth step, the combined standard uncertainty was calculated. The combined standard uncertainty *u*(*HV*) was obtained by combining the aforementioned uncertainty components, including *u*(*d*), *u*(*F*), *u*(*b*), *u*(*t*), and *u*(*r*), and is expressed as follows:

(7) $$u\left(HV\right)=\sqrt{{\left(\frac{\partial \left(HV\right)}{\partial d}\right)}^{2}u{\left(d\right)}^{2}+{\left(\frac{\partial \left(HV\right)}{\partial F}\right)}^{2}u{\left(F\right)}^{2}+u{\left(b\right)}^{2}+u{\left(t\right)}^{2}+u{\left(r\right)}^{2}}$$

where the calculated *u*(*HV*) values for the five regions (TA1, HAZ1, AZ61S, HAZ2, and SS304) are 3.076283 HV, 7.917202 HV, 6.047498 HV, 7.659427 HV, and 3.833969 HV, respectively. In the fifth step, the expanded uncertainty was calculated. The expanded uncertainty is given by the formula *U* = *k* × *u*(*HV*), where *k* = 2. In accordance with numerical rounding requirements, *U* must be rounded up. Therefore, the final expanded uncertainty *U* values for TA1, HAZ1, AZ61S, HAZ2, and SS304 are 7 HV, 16 HV, 13 HV, 16 HV, and 8 HV, respectively. In other words, for welded samples under a specific set of parameters, 95% of the possible microhardness measurement results fall within the intervals of ±7 HV, ±16 HV, ±13 HV, ±16 HV, and ±8 HV—expanded based on the average microhardness values of these five regions, respectively.

Based on the above uncertainty analysis, a further analysis was conducted on the effects of ring power and scanning frequency on microhardness. The variation patterns of microhardness under different parameters are relatively similar: as the measurement region extends from TA1 to SS304, the microhardness shows a trend of first decreasing and then increasing. This is because the hardness of the weld filling material AZ61S is lower than that of the base metals (TA1 and SS304) on both sides, and meanwhile, the base metals on both sides are supplied in a low cold-work hardening state. For the ring power, under different parameters, the average microhardness values of the five regions (TA1, HAZ1, AZ61S, HAZ2, and SS304) are (149.6 ± 3.4) HV, (131.1 ± 12.8) HV, (96.7 ± 13.2) HV, (297.5 ± 16.9) HV, and (310.4 ± 5.6) HV, respectively. For the scanning frequency, under different parameters, the average microhardness values of TA1, HAZ1, AZ61S, HAZ2, and SS304 are (149.7 ± 4.7) HV, (132.3 ± 11.7) HV, (92.4 ± 9.3) HV, (301.8 ± 15.6) HV, and (310.7 ± 5.3) HV, respectively.

For comparison, Xie welded 2 mm thick dissimilar butt joints of TA1 and SS304L using an IPG YLS-8000 fiber laser, with a 1 mm thick V sheet as the interlayer. The average microhardness values of the three regions (TA1, V, and SS304L) were (129.5 ± 3.5) HV, (77.8 ± 6.4) HV, and (203.3 ± 6) HV, respectively [28]. Zhao welded 1 mm thick butt joints of TA2 and SS301L using a pulsed Nd:YAG laser, and the microhardness results were as follows: with a 0.5 mm thick Cu sheet as the interlayer, the average microhardness values of TA2, Cu, and SS301L regions were (131.5 ± 11.4) HV, (175.8 ± 20.4) HV, and (173.8 ± 10.7) HV, respectively; without an interlayer, the average microhardness value of the weld zone was (310.2 ± 81.4) HV [29].

It can be seen from the above results that when AZ61S and V were used as interlayers, the average microhardness of the weld zone is lower than that of the base metals on both sides, and no hardening occurs in the weld. When Cu was used as the interlayer, or no interlayer was used, according to the analysis by the authors of Reference [29], the formation of Cu-Fe/Cu-Ti compounds or Fe-Ti compounds in the weld zone leads to higher hardness compared to the base metals on both sides, indicating hardening of the weld. From this perspective, the selection of AZ61S as the filling material in this study helps to prevent the occurrence of weld brittleness and hardening.

### 3.2. Bending Properties

**Figure 3:**
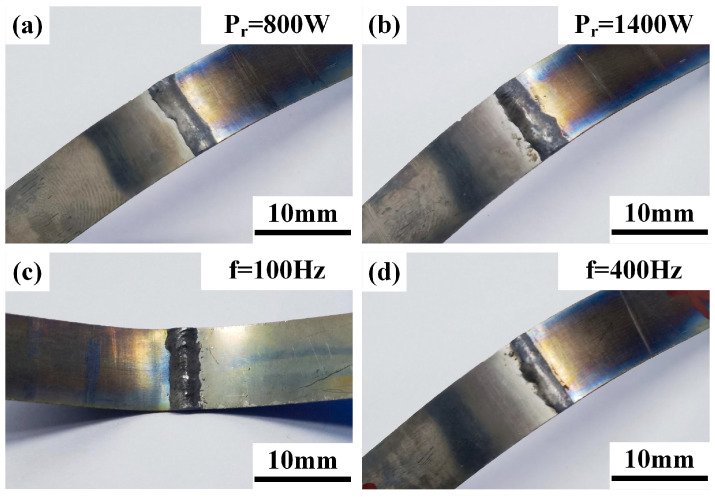
Surface morphology of weld horizontal bending, (**a**,**b**) typical ring power (when f = 300 Hz), (**c**,**d**) typical scanning frequency (when P_r_ = 1400 W).

**Figure 4:**
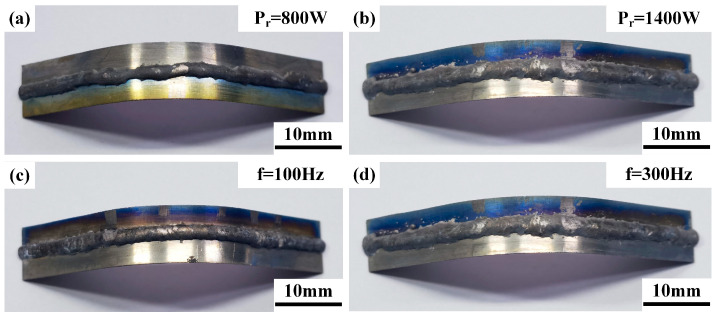
Surface morphology of weld axial bending, (**a**，**b**) typical ring power (when f = 300 Hz), (**c**，**d**) typical scanning frequency (when P_r_ = 1400 W).

Horizontal and axial bending performance tests were conducted on welded joints under different ring powers and scanning frequencies. With a bending radius of 30 mm, none of the specimens showed cracks until the bending indenter and the fixture automatically stopped due to interference, and the bending angle at this point was 108°. The weld surface morphologies of typical horizontal and axial bending specimens are shown in Figure 3 and Figure 4, respectively. The statistical results of bending performance are presented in Table 4.

### 3.3. Tensile Properties

**Figure 5:**
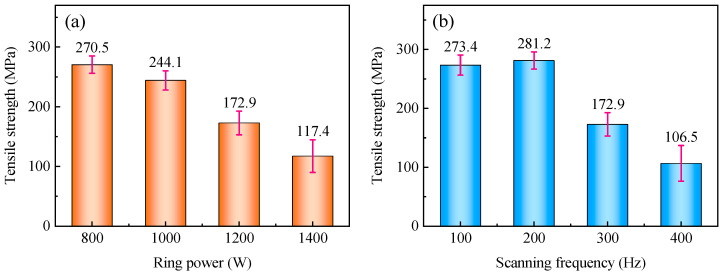
Effects of process parameters on tensile properties of welded joints: (**a**) ring power (when f = 300 Hz) and (**b**) scanning frequency (when P_r_ = 1400 W).

Effects of process parameters on tensile properties of welded joints are shown in Figure 5. When the ring power is in the range of 800–1000 W, or the scanning frequency is between 100 and 200 Hz, the tensile strengths of the welded joints are relatively high. Their average values are all greater than 80% of the tensile strength of AZ61S (approximately 240 MPa), and the maximum average value is 281.2 MPa, which is equivalent to 93.7% of that of AZ61S. As the ring power or scanning frequency further increases—specifically, when the ring power exceeds 1000 W, or the scanning frequency exceeds 200 Hz—the tensile strength of the joints shows a significant downward trend.

**Figure 6:**
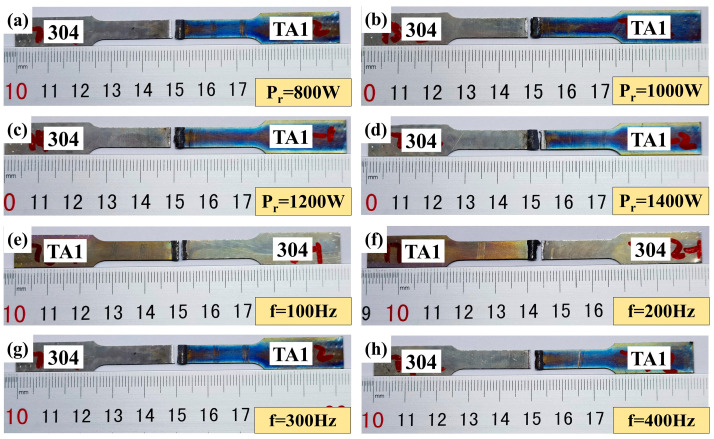
Morphology of typical tensile specimens after fracture: (**a**–**d**) ring power (when f = 300 Hz) and (**e**–**h**) scanning frequency (when P_r_ = 1400 W).

The appearances of typical tensile specimens under different ring powers and scanning frequencies are shown in Figure 6. A statistical analysis was conducted on the fracture positions of each specimen in Figure 6, and the results are presented in Table 5. It can be seen that among the eight sets of tensile specimens, seven sets fractured at the weld edge of the SS304/AZ61S interface, and one set fractured at the weld edge of the AZ61S/TA1 interface. This indicates that the fracture at the AZ61S/TA1 interface is an occasional phenomenon.

A comparative analysis was conducted on research results in similar fields. Xie performed butt welding on 2 mm thick TA1 and SS304L plates using a traditional non-ARM, non-scanning fiber laser [28]. With a 1 mm thick V sheet as the interlayer, the average tensile strength was 355 MPa, and the fracture occurred at the V/TA1 interface. The tensile strength of V ranges from 729 to 790 MPa [30], which is much higher than that of TA1. Thus, TA1 was used as the reference, and the average tensile strength of the specimens reached 83.5% of that of TA1 (425 MPa). Li welded 1.2 mm thick TA2 and SS304 butt joints using a scanning fiber laser, with Cu as the filling material [31]. When the thickness of the Cu sheet was 0.2 mm, 0.4 mm, and 0.6 mm in sequence, the tensile strengths of the welded joints were 145.7 ± 21 MPa, 260 ± 13 MPa, and 330 ± 19 MPa, respectively. All tensile specimens were fractured at the Cu/TA2 interface. The average tensile strength gradually increased with the increase in Cu sheet thickness, among which the maximum average value is 330 MPa, which was equivalent to 89.2% of that of Cu (370 MPa).

Gao welded 2 mm thick TC4 and SS304L butt joints using a traditional non-ARM, non-scanning fiber laser, with AZ31B welding wire of 1 mm diameter as the filling material [32]. When the laser power was 1.6 kW, 2.0 kW, 2.5 kW, 3.0 kW, and 3.5 kW in sequence, the tensile strengths of the joints were 48 ± 24.5 MPa, 126 ± 33.5 MPa, 221 ± 11.5 MPa, 186 ± 13 MPa, and 164.6 ± 14.5 MPa, respectively. Under the first three sets of parameters, the tensile specimens fractured at the SS304/AZ31B interface; under the last two sets of parameters, the fracture occurred at the AZ31B/TC4 interface. The average tensile strength first increased and then decreased with the increase in laser power, among which the maximum average value of 221 MPa was equivalent to 92.1% of that of AZ31B (240 MPa).

It can be seen from the above analysis that when the filling materials are V, Cu, and AZ31B magnesium alloy in sequence, the ratio of the tensile strength of the welded joints to the minimum tensile strength among the base metals and the filling material gradually increases. Considering that the filling materials are AZ61S and AZ31B magnesium alloys with similar compositions, the ratio of the maximum average tensile strength of the joints to the filling material is also improved by using the ARM scanning fiber laser (in this study), compared with the traditional non-ARM, non-scanning fiber laser (Reference [32]).

### 3.4. Fractography

**Figure 7:**
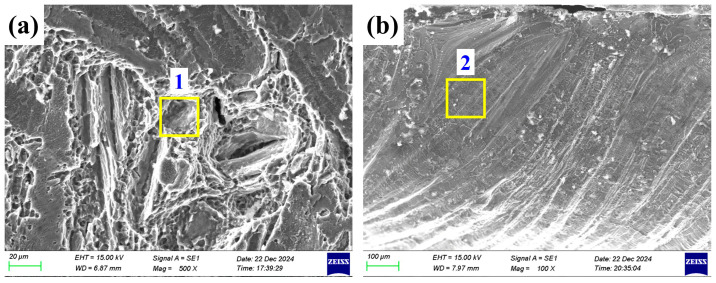
Fracture morphologies of typical tensile specimens: (**a**) SS304/AZ61S interface and (**b**) AZ61S/TA1 interface.

Typical specimens fractured at the SS304/AZ61S and AZ61S/TA1 interfaces in Figure 6 were selected to observe fracture morphologies by using SEM, as shown in Figure 7. Figure 7a shows that at the SS304/AZ61S interface, the fracture contains both dimple and cleavage morphologies, but dimples are the main feature. The size and distribution of the dimples are relatively uniform. Figure 7b indicates that at the AZ61S/TA1 interface, the fracture is dominated by cleavage morphology and exhibits the characteristic of incomplete fusion. As mentioned earlier, among the eight sets of tensile specimens, only one set fractured at the AZ61S/TA1 interface. Combined with Figure 7b, it can be seen that this is an accidental phenomenon. During the welding process, considering that the wettability of liquid Mg on Ti is better than that on Fe, the laser heat source and the filled magnesium welding wire were slightly shifted toward the 304 stainless steel as a whole. This leads to a low probability of poor fusion at the AZ61S/TA1 interface, which further results in the situation observed in this case.

Typical areas were selected from the fractures shown in Figure 7 to measure chemical compositions through EDS analysis. The results are presented in Table 6. At the SS304/AZ61S interface, the characteristic components of the fracture are dominated by Fe, Cr, and Ni (from SS304), followed by Mg, Al, Zn (from AZ61S), and Ti. Elements other than Fe, Cr, and Ni are mainly brought by the flow of the molten pool. At the AZ61S/TA1 interface, the contents of Mg, Al, Zn, and Ti in the fracture are relatively high, while the contents of Fe, Cr, and Ni (from SS304) are relatively low.

## 4. Discussion

### 4.1. Effects of ARM Laser on Mechanical Properties

Literature studies indicate that in dissimilar metal welding, FeAl is prone to form at the welded joints between stainless steel and magnesium alloy (corresponding to the SS304/AZ61S interface in this study) [33], and Mg_17_Al_12_ is likely to form at the welded joints between magnesium alloy and titanium alloy (corresponding to the AZ61S/TA1 interface) [34]. Mott proposed that Poisson’s ratio (*ν*) can be used to evaluate the brittleness of materials [35]: when the *ν* value is less than 0.26, the material exhibits brittleness; when the *ν* value is greater than 0.26, the material exhibits ductility. The calculation formula for the *ν* value is as follows:

(8) $$\nu =\frac{3B-2G}{2\left(3B+G\right)}$$

where *B* is the bulk modulus, and *G* is the shear modulus. For FeAl, *B* is 138.3 GPa, and *G* is 103.03 GPa [36]; for Mg_17_Al_12_, *B* is 48.1 GPa, and *G* is 31.6 GPa [37]. Calculations show that the *ν* value of FeAl is 0.20 and that of Mg_17_Al_12_ is 0.23, both of which are less than 0.26, indicating that FeAl and Mg_17_Al_12_ are brittle phases. Since the *ν* value of FeAl is approximately 13% lower than that of Mg_17_Al_12_, FeAl exhibits greater brittleness. That is to say, in mechanical property tests, if fracture failure occurs, it will preferentially occur at the SS304/AZ61S interface that is rich in FeAl. This explains why most tensile specimens are fractured at the SS304/AZ61S interface. In the following contents, the SS304/AZ61S interface will be used as an example to discuss the effects of parameter changes in the ARM laser and laser scanning motion on mechanical properties.

**Figure 8:**
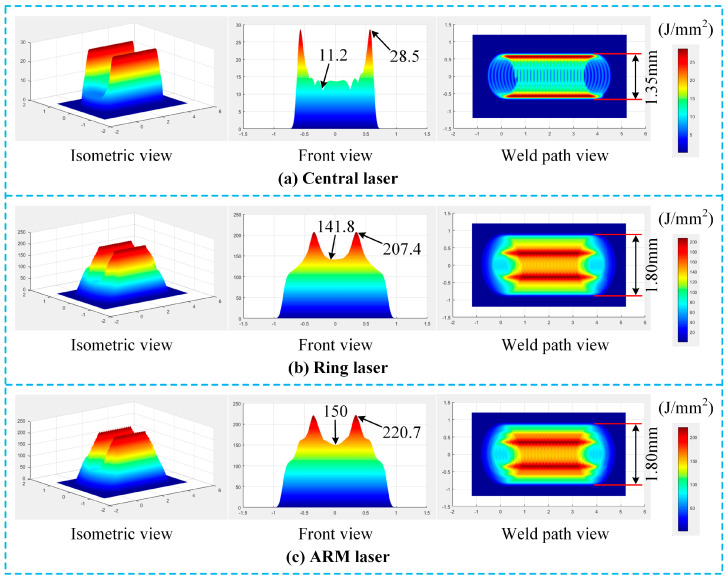
Heat source energy distribution along the welding path (when P_c_ = 800 W, P_r_ = 1400 W, and f = 300 Hz), (**a**) central laser, (**b**) ring laser, and (**c**) ARM laser.

The biggest difference between the ARM laser and traditional lasers lies in the addition of an annular laser beam around the periphery of the central laser. The energy density distributions of the central laser (*I_c_*) and the ring laser (*I_r_*) can be calculated by Equations (9) and (10), respectively [13], while the energy density distribution of the ARM laser (*I*) can be calculated by Equation (11).

(9) $$I_{c}=\frac{3\eta P_{c}}{\pi R_{c}^{2}}\exp\left(\frac{-3r^{2}}{R_{c}^{2}}\right)$$

(10) $$I_{r}=\frac{3\eta P_{r}}{\pi \omega^{2}}\exp\left(\frac{-3{\left(r-R_{r}\right)}^{2}}{\omega^{2}}\right)$$

(11) $$I=I_{c}+I_{r}$$

where in these equations, *η* is the absorption coefficient of the material, *P_c_* and *P_r_* represent the power of the central laser and ring laser, respectively, *R_c_* is the spot radius of the central laser, *R_r_* is the radial distance from the position of maximum energy density in the ring laser beam to the center of the circle, *ω* is half the width of the ring laser, and *r* is the radial position within the laser beam. For materials with different thermophysical properties, the absorption coefficient *η* in Equations (9) and (10) integrates the effects of thermal conductivity, specific heat capacity, and laser absorptivity. This allows the model to be extended to materials with different thermophysical characteristics and demonstrates its wide applicability. According to the scanning laser energy deposition model proposed by Meng [38], the spiral trajectory of a circular scanning laser can be decomposed into movements along the x and y directions, as shown in Equation (12):

(12) $$\left\{\begin{array}{l} x\left(t\right)=x_{0}+vt+A\sin\left(2\pi ft+\varphi_{x}\right)\\ y\left(t\right)=y_{0}+A\cos\left(2\pi ft+\varphi_{y}\right) \end{array}\right.$$

where *x*(*t*) and *y*(*t*) represent the spot position, and *r*^2^ = [*x*(*t*)]^2^ + [*y*(*t*)]^2^ (*r* has been defined in Equations (9) and (10)), *x*_0_ and *y*_0_ are the initial coordinates of the spot, *v* denotes the welding speed, *A* is the scanning amplitude, *f* is the scanning frequency, and *φ_x_* and *φ_y_* are the initial phase angles in the x and y directions, respectively. By substituting Equations (9), (10), and (12) into Equation (11) and by integrating over time, Equation (13) is obtained. The energy distribution state along the welding path within the time span *T_p_* can be acquired through Equation (13), as shown in Figure 8.

(13) $$E\left(x,y,t\right)=\int_{0}^{T_{p}}I\left(x,y,t\right)dt$$

As can be seen from Figure 8a, during the scanning process of the central laser, its energy distribution exhibits a wavy characteristic with higher energy at the edges and lower energy in the middle. The measured energy density at the edge wave crests is 28.5 J/mm^2^, while that at the central wave troughs is 11.2 J/mm^2^; the ratio of wave troughs to wave crests is 39.3%, and the measured weld width is 1.35 mm. Figure 8b shows the energy distribution of the ring laser. During scanning, its energy distribution also presents a wavy pattern with higher energy at the edges and lower energy in the middle. The energy density at the edge wave crests is 207.4 J/mm^2^, that at the central wave troughs is 141.8 J/mm^2^, the ratio of wave troughs to wave crests is 68.4%, and the weld width is 1.80 mm. Figure 8c displays the ARM laser, which combines the central laser and the ring laser. Its energy density at the edge wave crests is 220.7 J/mm^2^, that at the central wave troughs is 150 J/mm^2^, the ratio of wave troughs to wave crests is 68.0%, and the weld width is 1.80 mm.

By comparing Figure 8c with Figure 8a, it can be observed that, compared with the central laser alone, the ARM laser (as a whole) has the ratio of wave troughs to wave crests increased by 29.1 percentage points (a 74% improvement), and the weld width increased by 33.3%. The central laser is a Gaussian beam, whose spot pattern is very close to that of the traditional non-ARM laser. In other words, compared with the traditional non-ARM laser, the ARM laser has significantly improved energy distribution uniformity and a notably larger molten pool volume.

**Figure 9:**
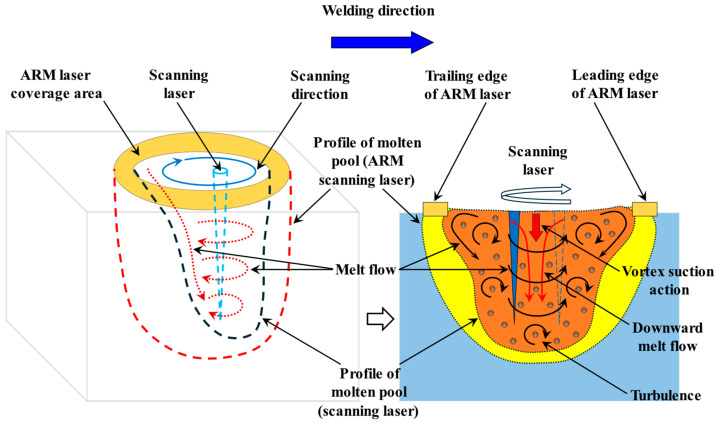
Influence of ARM laser on molten pool flow.

Figure 9 provides further analysis of this phenomenon. Compared with the non-ARM laser, the ARM laser has a significantly larger heat-affected area, which leads to the expansion of the molten pool volume. Combined with the observations from Figure 8, the ARM laser exhibits a more uniform energy distribution. Its temperature gradient during molten pool solidification is smaller than that of the non-ARM laser, and thus its solidification rate is lower. From Figure 9 can also be seen that, when viewed from the cross-section along the welding direction, if the annular beam in front of the central beam is referred to as the “leading edge of the ARM laser,” and the part behind the central beam as the “trailing edge of the ARM laser,” then the leading edge of the ARM laser mainly functions as preheating. It raises the temperature of the unmelted base metal ahead of the welding zone and enhances the metal’s laser absorption capacity. In contrast, the trailing edge of the ARM laser primarily maintains the temperature of the molten pool and exerts a certain degree of remelting effect on the newly formed solid–liquid interface.

When the trailing edge of the ARM laser remelts the newly formed solid–liquid interface, it breaks the just-solidified FeAl interface layer and creates several micro-gaps at its weak points. At this time, Fe atoms on the surface of the stainless steel base metal continuously dissolve into the molten pool through these micro-gaps. With the high-frequency scanning of the laser, the molten pool after multiple remelting cycles has a higher Fe concentration compared to an unremelted molten pool. This leads to a thicker FeAl interface layer during growth, which in turn causes a decrease in the mechanical properties of the welded joints. Therefore, when the ring power exceeds 1000 W, its remelting effect on the stainless steel matrix becomes excessively strong. This results in an excessively high concentration of Fe in the molten pool, and the subsequent increase in the thickness of the FeAl interface layer ultimately leads to a significant drop in tensile strength.

### 4.2. Mechanism of Laser Scanning on Tensile Property Variations

There is an agitation effect in the molten pool of scanning laser welding. If the molten pool is regarded as a stirred tank and the laser keyhole with high-speed scanning is treated as an agitator, then according to fluid mechanics and melt agitation theory, the liquid flow state can be characterized by the agitation Reynolds number *Re*. Its formula is as follows [39]:

(14) $$R_{e}=\frac{\rho ND^{2}}{\mu }=\frac{\rho f{\left(2A\right)}^{2}}{\mu }$$

where *ρ* is the fluid density, *N* is the agitator rotational speed, *D* is the agitator diameter, *µ* is the viscosity of molten metal, *f* is the scanning frequency, and *A* is the scanning amplitude. Generally, when *Re* is less than 1000, the molten flow inside the molten pool exhibits a laminar flow pattern; when *Re* is greater than 1000, the molten flow transitions to a turbulent flow pattern.

**Figure 10:**
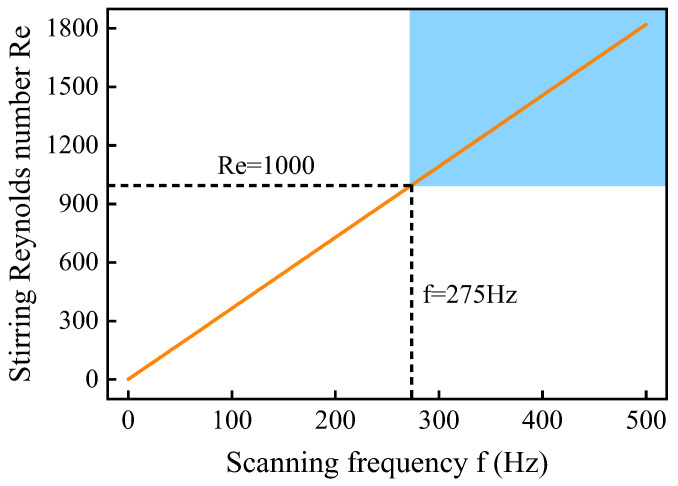
Relationship between laser scanning frequency *f* and molten pool agitation Reynolds number *Re*.

According to Equation (14), the Reynolds number *Re* values under different laser scanning frequencies can be calculated. For the liquid Mg molten pool, *ρ* is taken as 1.74 × 10^3^ kg/m^3^ and *µ* as 6.89 × 10^−4^ Pa·s [40]. As shown in Figure 10, when *f* ≤ 275 Hz (*Re* ≤ 1000), the molten pool flow is in a laminar state. When *f* > 275 Hz (*Re* > 1000), the molten pool flow transitions to a turbulent state. During the welding process, the molten flow induced by the scanning laser beam continuously scours the newly formed solid–liquid interface. When this interface is still relatively weak, the intense scouring effect of turbulence creates several micro-gaps in the interface layer. This causes Fe atoms on the surface of the stainless steel to continue dissolving into the molten pool through these micro-gaps. Therefore, the molten pool in a turbulent state has a higher Fe concentration than that in a laminar state, resulting in a thicker FeAl interface layer and thus poorer mechanical properties. Reflected in tensile strength, when the scanning frequency is equal to or greater than 300 Hz, the tensile strength of the welded joints decreases significantly.

## 5. Conclusions

(1) The microhardness distribution shows a trend of being higher in the base metals on both sides and lower in the middle filling area, which is related to the filling material. Compared with the case where Cu is used as the filling material, the selection of AZ61S as the filling material helps prevent the occurrence of weld brittleness and hardness. Horizontal and axial bending test results of the welded joints indicate that the bending angle of all specimens can reach 108°, and no cracks appear until the bending testing machine stops.
(2) When the ring power is in the range of 800–1000 W, or the scanning frequency is between 100 and 200 Hz, the average tensile strengths of the welded joints are all higher than 80% of that of AZ61S (approximately 240 MPa); the maximum average value (281.2 MPa) can reach 93.7% of that of AZ61S. As the ring power or scanning frequency further increases, the tensile strength of the joints shows a decreasing trend. Compared with the cases where V, Cu, and AZ31B were used as the filling material, the ratio of the tensile strength of the welded joints to the minimum tensile strength among the base metals and the filling material is increased.
(3) The remelting effect of the trailing edge of the ARM laser when its energy is relatively high, or the scouring of the turbulent molten flow induced by the scanning laser beam, will damage the newly formed solid–liquid interface and create several micro-gaps at the weak points of the FeAl interface layer. Fe atoms on the surface of the stainless steel continuously dissolve into the molten pool through these micro-gaps, which increases the Fe concentration in the molten pool. Consequently, the grown FeAl interface layer becomes thicker, leading to a decrease in the mechanical properties of the welded joints.

## Figures and Tables

**Table 1:** Chemical compositions of the base metals and filling material (wt.%).

Material	Ti	Fe	Cr	Ni	Mg	Al	Zn	Cu
TA1	Bal.	≤0.20	-	-	-	-	-	-
SS304	-	Bal.	18.0–20.0	8.0–11.0	-	-	-	-
AZ61S	-	≤0.005	-	≤0.005	Bal.	5.5–6.5	0.5–1.5	≤0.05
Material	C	N	H	O	Si	Mn	P	S
TA1	≤0.08	≤0.03	≤0.015	≤0.18	-	-	-	-
SS304	≤0.08	-	-	-	≤1.00	≤2.00	≤0.045	≤0.030
AZ61S	-	-	-	-	≤0.10	0.15–0.4	-	-

**Table 2:** Mechanical properties of the base metals and filling material.

Material	TA1	SS304	AZ61S
Microhardness (HV)	≤150	≤310	≤100
Tensile strength (MPa)	426 ± 20.9	≥780	280–300

**Table 3:** Welding parameters in the experiment.

Welding Parameters	Variation Ranges	Baseline Values
Central power, P_c_ (W)	800	800
Ring power, P_r_ (W)	800–1400	1400
Scanning frequency, f (Hz)	100–400	300
Scanning amplitude, A (mm)	0.6	0.6
Welding speed, v (m/min)	2.5	2.5
Wire feed rate, v_w_ (m/min)	6.0	6.0
Defocus distance, h (mm)	0	0

**Table 4:** Statistical results of bending performance (N means “no”).

Type	Parameters	Bending Angle (Horizontal)	Whether Cracks Occur	Bending Angle (Axial)	Whether Cracks Occur
Ring power (when f = 300 Hz)	P_r_ = 800 W	108°	N	108°	N
	P_r_ = 1000 W	108°	N	108°	N
	P_r_ = 1200 W	108°	N	108°	N
	P_r_ = 1400 W	108°	N	108°	N
Scanning frequency (when P_r_ = 1400 W)	f = 100 Hz	108°	N	108°	N
	f = 200 Hz	108°	N	108°	N
	f = 300 Hz	108°	N	108°	N
	f = 400 Hz	108°	N	108°	N

**Table 5:** Statistics on fracture positions of tensile specimens.

Parameters (when f = 300 Hz)	P_r_ = 800 W	P_r_ = 1000 W	P_r_ = 1200 W	P_r_ = 1400 W
Fracture positions	SS304/AZ61S	SS304/AZ61S	SS304/AZ61S	AZ61S/TA1
Parameters (when P_r_ = 1400 W)	f = 100 Hz	f = 200 Hz	f = 300 Hz	f = 400 Hz
Fracture positions	SS304/AZ61S	SS304/AZ61S	SS304/AZ61S	SS304/AZ61S

**Table 6:** EDS analysis results of fracture areas #1 and #2.

Area	Type	Chemical Compositions (wt.%)						
		Mg	Ti	Cr	Fe	Ni	Zn	Al
#1	SS304/AZ61S interface	18.53	2.26	19.26	29.6	18.95	3.72	7.68
#2	AZ61S/TA1 interface	31.39	25.29	3.37	8.19	5.24	9.13	17.39

## Data Availability

The original contributions presented in this study are included in the article. Further inquiries can be directed to the corresponding author.
